# Representations of influenza and influenza-like illness in the community - a qualitative study

**DOI:** 10.1186/1471-2296-14-15

**Published:** 2013-01-24

**Authors:** Christine Cedraschi, Laurence Saya, Patrick Klein, Marie-France Bordet, Fabrice Carrat

**Affiliations:** 1 Division of Clinical Pharmacology and Toxicology, Multidisciplinary Pain Centre, Geneva University Hospitals & University of Geneva, Geneva, Switzerland; 2 Division of General Medical Rehabilitation, Geneva University Hospitals & University of Geneva, Geneva, Switzerland; 3 Altius Pharma CS, Paris, France; 4 Vision Critical Communication SAS, Paris, France; 5 Medical Department, Boiron, Lyon, France; 6 APHP, Hôpital Saint-Antoine, Unité de santé publique, F-75012, Paris, France; 7 UPMC Université Paris 06, UMR-S 707, F-75012, Paris, France; 8 INSERM U707, F-75012, Paris, France

## Abstract

**Background:**

There is little information regarding lay-people's representations of influenza and influenza-like illness in their day-to-day lives. An insight into these views may aid our understanding of community attitudes regarding official recommendations for its prevention.

**Methods:**

This was a qualitative research. Semi-structured face-to-face interviews were conducted with 40 French participants from the community, and from five different locations. Questions elicited the participants' representations of onset of flu and influenza-like illness, as well as their views on what can/should be done to deal with symptoms and their personal experience with flu and flu-like symptoms.

**Results:**

Thematic content analyses allowed us to identify five main themes: the presence of a clear continuum between influenza-like illness and flu; a description of flu as a very contagious disease; flu as being benign, except in "frail people", which the respondents never considered themselves to be; interruption of daily activities, which could be considered pathognomonic for influenza for most subjects; self-medication as the main current practice, and requests for healthcare mainly to confirm an auto-diagnosis.

**Conclusions:**

There was a large homogeneity in the representation of flu. There was also a gap between people's representations (i.e., a continuum from having a "cold" to having "influenza") and scientific knowledge (i.e., a distinction between "true" influenza and influenza-like illnesses based on the existence of a confirmatory virological diagnosis). This gap raises issues for current campaigns for flu prevention, as these may not be congruent with the representation of flu being responsible for interrupting daily activities while also being seen as a non-severe disease, as well as the perception that flu is only a risk to "frail people" though no participants considered themselves to be "frail".

## Background

Influenza is a recurrent public-health issue, and there is considerable information on influenza from an epidemiological point of view. Community subjects’ knowledge of preventive measures has dramatically increased since the occurrence of the H1N1 pandemic, although there is still a gap between scientific information and every-day life [1]. Little information is available regarding lay people’s representations of influenza and of how it may affect them in their day-to-day lives.

An English study, conducted in the 1970s, showed that patients think that colds occur when vulnerable parts of the body (e.g., feet, head, chest) are exposed to cold temperatures, humidity, or cold air currents [2]. In a US study, parents tended to find an association between the weather changes and the incidence of their children's colds [3]. Although information is available regarding people’s knowledge about avian flu and its prevention, there is little information on how preventive measures, e.g., wearing a mask or the need for social distance, are endorsed by community subjects [4].

Flu-vaccination issues provide some information concerning people’s views. A qualitative study in people aged > 65 years demonstrated that, for these subjects, the risk of contracting flu was considered very low, with no consequences of the vaccination on this probability. Also, if they caught flu, they did not expect any serious consequences [5]. This feeling of the unlikeliness of contracting this disease was also reported in an Australian study that focused on the H1N1 pandemic [6]. Qualitative studies show that, in a non-pandemic context, few people expressed favorable opinions about control behaviors or were ready to adopt avoidance actions, and they questioned the effectiveness of vaccination, even in a pandemic context [7,8]. As Gray *et al.* stressed [9], “It is important to accommodate the fact that disbelief in the effectiveness of measures can result in people failing to act and developing distrust of sources of information”. Similarly, a French qualitative study, conducted on a group of patients with cystic fibrosis, a high risk population for severe flu infection, emphasized the role of information sources in these patients in deciding to accept or refuse the H1N1 vaccine. Those who refused the vaccine mentioned receiving multiple and indecisive information, whereas those who accepted the vaccine reported having received unequivocal advice from their healthcare provider [10]. Globally, results from these qualitative studies emphasize that the public need to receive transparent and factual information about the specific actions to be take, and this should be provided by people they can trust [9].

These data question the representations that people have about this disease and the congruence of these representations versus scientific definitions. Influenza (or flu) is a contagious viral infection caused by influenza viruses, which mainly affects the nose, throat, bronchi, and, occasionally, the lungs. The combination of fever or other systemic symptoms, plus respiratory signs, defines an influenza-like illness (ILI). Influenza viruses can cause ILI, but not always: ~30% of influenza infections are not associated with any symptoms, and another ~30% will not cause a fever. Thus, only ~30% of influenza infections correspond to ILI [11]. In contrast, not all ILIs are caused by influenza viruses: many other respiratory pathogens can cause the same type of illness. In temperate countries, the likelihood of having the influenza virus in a patient with an ILI varies from 0% (when no influenza virus is circulating in the community, e.g., in summer) to 60–70% when there is intense circulation (e.g., during seasonal epidemics). Of note, vaccination or specific antiviral therapies will only prevent or treat infections and illnesses (mild or severe) caused by the influenza virus: i.e., they are not effective against all ILIs.

Peoples' representations consist of socially constructed and shared knowledge based on experiences and models of thoughts spread via education and social communication [12,13]. As a form of practical knowledge, representations help understanding and explaining our universe. Patients' representations of flu are an important aspect of the patients' decision-making processes regarding treatment and their perceptions of treatment [14]. These prior elements influence the way that people organize new information (i.e., whether to incorporate it with existing knowledge or to discard it) [13].

Anthropological studies suggest two groups of representations of the disease in the public health and social field [15]. The "majority model" views the disease as an exogenous entity that enters the body of an individual who carries no responsibility or control over the process. Healing is viewed as suppressing a hostile condition that must be annihilated. This "majority model" mainly draws from the biomedical model which considers the etiology and symptoms of the disease when choosing a treatment. The second model considers disease as an endogenous entity and healing as a regulating activity that takes into account, rather than opposes, the symptoms of the disease. This model stresses the role of the patient in curing him- or herself.

Taken together, these elements suggest a possible gap between scientific and lay knowledge, with both forms of knowledge having different aims. While scientific knowledge describes general patterns of disease common to all individuals and statistically appraisable, lay knowledge tends to personalize the illness by including the individual’s suffering and then contextualizes it [16]. In the scientific model, flu and ILI dictate various strategies of prevention and treatment according to the patient's medical history, age, etc. However, lay people’s representations regarding flu and ILI, as well as attitudes and behaviors related to its symptoms, have received little research. This issue is of importance as it is a seasonal problem, and public-health authorities and clinicians involved in day-to-day management of disease have to elaborate and try to implement strategies directed at informing various groups within the population about the disease, its risks, and possible preventive interventions.

Based on these observations, we carried out a qualitative analysis designed to improve our understanding of people’s representations of flu and ILI in France. Indeed, a better insight into the sorts of views people hold about flu and ILI, as well as how they plan to cope with its symptoms, is particularly relevant as influenza is a highly recurrent disease.

## Methods

### Study design

We chose a qualitative study design as data about flu and ILI representations in the community are lacking so far. Face-to-face semi-structured interviews were thought to be the best setting to retrieve information. On the basis of these interviews, thematic content analysis allowed us to identify categories and themes.

### Participants

Because flu/ILI can affect the general population, the selection of participants took into account the diverse French population. Despite the qualitative methodology used for this study, we used the “quotas” method to determine the choice of participants: this took into account gender, age (18–34, 35–54, 55–64, 65–74 years), socio-professional group, place of abode (urban/rural, northern/southern area), in order to represent the whole French population as much as possible.

Forty subjects, aged between 18 and 74 years, were recruited to participate in 1-hour individual interviews. They lived in five French cities (Paris, Lyon, Nîmes, La Rochelle, Besançon), which differ in size and environment. Participants were recruited via telephone, by random, from telephone directories. A screening questionnaire was used to identify socio-demographic characteristics, and they were then asked if they would participate in the study. In order to obtain a better spontaneous representation and to avoid biases that could be linked to the preparation of the interview, participants were informed that the interview had to do with general health. About 80% of the recruited individuals agreed to participate. Because of the "quota" method used in the sampling, those who refused were replaced by individuals with the same characteristics in terms of gender, age, socio-professional group, and place of abode. Those who refused did so mainly because of time contingencies. Respondents were given a small fee (35€) for participating. The recruited sample allowed us to include the whole range of possible responses and to achieve theoretical "saturation" [17], i.e., the point where no new categories emerged from the analysis of the interviews.

### Data collection

Participants were questioned using face-to-face semi-structured interviews [18] between January to February 2012. Open-ended questions elicited the patients’ representations of the onset of flu and ILI, i.e., their views on the initial symptoms and how they develop. The interview also investigated their views on what can/should be done to deal with symptoms and their personal experience with flu and flu-like symptoms.

An interview guide (Table 1) was developed, which included general topics on flu and ILI: the topics were not addressed in a fixed order. The interviewers were flexible and responsive to issues as that arose regarding the participants’ views so that these could be further explored where appropriate. However, the opening questions always revolved around the participants’ descriptions of their views about flu and ILI. As the interview progressed, further issues on alarm signals, diagnosis, and seeking help were addressed. Respondents were prompted to give their own opinions, and were told that their personal points of view about the disease were of interest and that there were no right or wrong answers. Qualitative methods were chosen, as the aim was to access the range of participants' views and to record their individual ways of thinking [19,20]. This is in line with the use of qualitative data in health research to provide a perspective that goes beyond purely quantitative information [21]. Two experienced interviewers conducted the interviews, which lasted 45–60 minutes each.

**Table 1:** Interview guide

**Topics**	**Examples**
General representations	Which words or images do you spontaneously associate with flu?
	How would you describe flu? (respectively, flu-like symptoms)
	Which words do you use to describe it?
Personal experience	Have you ever had the flu?
	Can you describe this experience?
Alarm signals and help-seeking	What were the symptoms that made you think that you might have the flu?
	What did you do when you thought you might have the flu?
	Was there anything you could do to reassure yourself? Find relief?
Prevention	Can flu be prevented?
Information needs	Do you feel well informed about flu?
	What additional information do you need?

Data were de-identified to ensure confidentiality. There was no request for ethical committee approval as, in accordance with French Law, such studies are not within a biomedical research category (no patients, no treatments). All subjects were informed of the goals and design of the study and agreed to participate.

### Analysis

The interviews were recorded and transcribed. Transcripts were checked against audio files for accuracy and compared with scribed notes taken during each interview. The transcripts were analyzed using a manual data-indexing technique to identify key themes [22]. The qualitative analysis began with the two researchers reading the transcripts individually. Analysis then continued throughout data collection and the coding process, using the constant-comparative method, which consists of analyzing the interviews by comparing one response with earlier observed responses [23]. This was followed by comparisons between the transcripts, which were then used to establish analytical categories.

These categories served as the basis for a final grid, which was then used independently by the two researchers to analyze the transcripts to maximize theoretical sensitivity and rigor [24]. Using patient-generated data via the interviews and verification of interpretation by the two researchers allowed assessment to be trustworthy [20].

## Results

### Characteristics of the participants

The characteristics of the forty participants are described in Table 2. Twenty-two women and 18 men participated; their age ranged from 21 to 72 years; 22 were from an urban area; and the sample included a variety of professional qualifications.

**Table 2:** **Characteristics of the study's participants (*n*=40)**

**Parameter**		**Results**
Age (years)	Mean ± standard deviation	44.9 (14.5)
	Median	43.5
Gender	Women: *n* (%)	22 (55%)
	Men: *n* (%)	18 (45%)
Living area	Urban: *n* (%)	22 (55%)
	Semi-urban: *n* (%)	11 (27.5%)
	Rural: *n* (%)	7 (17.5%)
Occupation	A non-qualified workers or employee: *n* (%)	9 (22.5%)
	Qualified worker or employee: *n* (%)	8 (20%)
	Managerial staff: *n* (%)	7 (17.5%)
	Professionally inactive (unemployed, retired, housewife/husband): *n* (%)	15 (37.5%)

### Representations of flu and flu-like symptoms

The interview-reviewing process identified five major themes: identification of a continuum; the major consequences to daily living; contagiousness of the disease; the benignness of flu except in frail people; and a request for healthcare.

### Identification of a continuum

**Figure 1:**
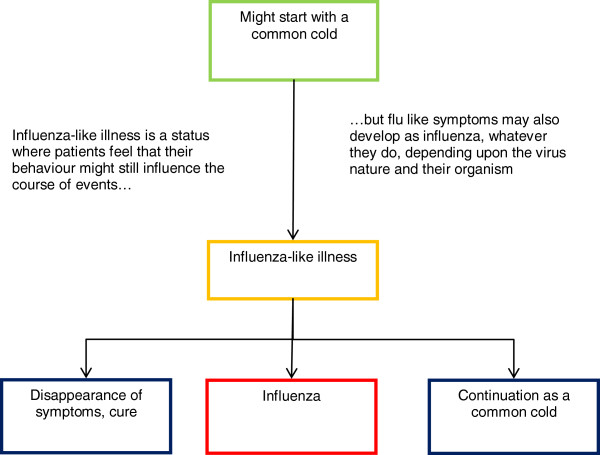
“Influenza-like illness” according to participants.

Most participants expressed the idea of a continuum from “cold” to “influenza (flu)” including “influenza-like illness” in between. The term “influenza-like illness”(ILI) identified a status that was understood by all participants, but nearly never used in real life. According to the participants, this expression was rather part of the medical or pharmaceutical terminology, and labels a general bad physical shape, a status that was not yet exactly “influenza” itself, but very near it. This term referred to a sort of a border, an undefined no man’s land where clear trouble could develop or symptoms could disappear. For nearly all participants, “ILI” was seen as the premises of flu: symptoms are the same, they differ by severity only, last a few days (less than three), and allow for maintaining daily activities. *A contrario*, the “flu” was identified as a specific disease. It is a medical issue, with well-defined contours. It was described as characterized by a longer duration (over 5 days), symptoms of higher intensity, and by a mandatory interruption in daily activities, with a need for confinement to bed (see Figure 1), and a clear-cut diagnosis possibly provided by the physician.

Both the identification of this continuum and the description of its phases were very similar across the different groups of participants, providing a homogeneous representation of the illness and its symptoms. Table 3 provides quotes illustrating this difference in intensity between ‘flu’ and ‘ILI’.

**Table 3:** Participants' descriptions of influenza-like illness and flu

**Influenza-like illness**	**Flu**
Influenza-like illness, it is when I feel miserable, with light fever, I do not feel good [respondent 27, 47-year-old man]	Flu, I have got a temperature of 40°C, I sweat, I am not able to stand, I am not able to eat anything [respondent 27, 47-year-old man]
I would say a general tiredness, chills, having a common cold [respondent 6, 26-year-old woman]	Flu is more violent, it is fever, it is when I cannot stand up. It is not frequent for me, but it happened to me [respondent 19, 36-year-old woman]
With influenza-like illness, it is still possible to take one’s car and to move [respondent 3, 33-year-old woman]	Chills and pain everywhere. And after, I do not feel good, and just wish to stay in bed [respondent 6, 26-year-old woman]
Influenza-like illness, I could say cold, throat pain, with no aches. Cold, aches and little fever [respondent 32, 55-year-old woman]	It is when we cannot move, when the temperature is so high that I do not get out of bed [respondent 3, 33-year-old woman]
Influenza-like illness I would say it may not yet be broken out, you feel something is to happen. Flu-like symptoms, it is before flu [respondent 14, 70-year-old man]	It is 38°C. Flu it is actually bed ridden, you do not move [respondent 32, 55-year-old woman]
I do not use, I could vaguely see what it is, it is between two things. It is worse than a cold, and not exactly flu. For me, either I have a cold, or I have the flu. There are only two. [respondent 12, 25-year-old man]	I would say that what is common between all people with flu is that they were completely exhausted and most of the time, they were staying in bed [respondent 31, 43-year-old man]

As for all participants «ILI» was seen as attenuated “flu” symptoms, only flu representations will be presented hereby.

### In real life, flu interrupts daily-living activities

A cluster of symptoms were associated with flu, including fatigue as the main symptom, ranging from tiredness to exhaustion, and from lack of energy to immobilization, and to bed confinement. Pain, aches, and, especially, headaches, were frequently cited, followed by fever, chills, and shivers. Flu was particularly perceived as preventing daily activities, including being unable to work or to take care of relatives. Thus, it was described as kind of isolation that could not be escaped, and which lasted as long as the symptoms did.

All the respondents (except five), independently of their gender, age, or work status, mentioned the interruption to daily activities (i.e., whether they were professionally active or not, and whether they had an independent or dependent work status). The doctor might then be asked to visit to confirm diagnosis and to provide a certificate to permit an interruption from work:

“*[The doctor] will suggest it spontaneously, I don’t believe I need to ask him to prescribe me a sick-leave certificate because, in general, you’re physically no longer able to work; with a fever as high as 39°C you’re bed-ridden!*” [respondent 15, man, 43 years old].

Interruption of daily activities was often the axis point where the continuum between having a “cold” to having “flu” was reached. The need for a sick-leave certificate was further confirmation, and the acknowledgement that it was the person’s right to stay at home because of the flu:

“*If it stalls, I stay put. If it really worsens, I call the doctor and I go visit him… but only if it really worsens! […] Last time I got the flu, I had to take a week off. I think I called my boss to tell him I couldn’t go to work because I had the flu. If he asked me to, I probably called my doctor but just to get the sick certification*” [respondent 5, woman, 56 years old]*.*

Flu was perceived as a disease that sapped all energy, and for which there was no miracle cure. Rest and confinement to bed seemed not only the right thing to do, but also the only possible response due to the flu-related fatigue and loss of drive.

“*Not willing to do anything which requires energy, taking care of my children for instance when I have got the flu, I am really not willing to work, for sure. Anything that requires a physical effort*” [respondent 19, see Table 3].

Flu was also seen to impact on relationships with family and friends, with a need for solitude in a noiseless environment uniformly described by participants.

“*I do not request anything, not willing to eat, not willing to drink, only one desire, staying in bed and sleeping*” [respondent 3, see Table 3].

### A very contagious disease but that is not easily caught

Flu was not only considered to cause exhaustion and to interrupt daily life, but also as a contagious disease. Around three-quarters of participants declared they had contracted flu at least once in their life. However, most stated that they had not had flu more than once or twice in their lives.

“*It’s contagious, it’s a virus, it can be through someone who’s infected or in the air… […]. Me I’d say I’m usually very sensitive for all colds and this kind of things, but for the flu, in all and for all, I’ve had it two or three times… I see around me, people will get colds, sneeze, cough, but the flu really, I don’t think it’s so easy to catch it… maybe older people but otherwise no…*” [respondent 31, man, 43 years old].

These descriptions, depending upon previous personal and familial flu histories, were very homogeneous between participants. The participants’ views regarding different aspects of flu (cause, contagiousness, mode of transmission, etc.…) were globally congruent with scientific knowledge, built on family transmission, experience, recurring information in the media during the flu period, and confirmation of auto-diagnosis by a medical diagnosis. Nearly two-thirds of participants identified a virus as responsible for influenza. If the mode of transmission was not immediately obvious, after repetition of the question, respondents suggested potential forms of transmission, such as through sneezing, spluttering, kissing, or through direct contact via hands or contaminated objects.

When asked about the possibilities of preventing flu or ILI, some respondents mentioned several methods, including the use of homeopathic medicines and/or included this prevention in a larger context that emphasized a healthy lifestyle. A number of participants mentioned behaviors related to their view about how flu was transmitted, the contagiousness of flu, in particular washing hands and avoiding close contact (e.g., kissing people, shaking hands, or using public transportation). However, many also indicated that these measures might not be realistic in their everyday lives, in particular, avoiding contact.

Although transmission is easy, catching flu requires specific conditions, as this participant indicated: “*It’s infectious […] If you cough on me, there are indeed more chances that I’ll catch it… Then also, when the air is contaminated, like in the train where the air is not often recycled… then yes, there are more chances… but I think that one also has to be in a specific condition that is, for example, to be physically exhausted to really become sick*” [respondent 7, man, 28 years old]. These specific conditions paralleled the representation of flu as a problem for frail people.

### A benign disease except in frail people

Although reported as interrupting daily-life activities and being highly contagious, flu was also seen as a benign disease. When participants were asked about the “family” of diseases in which flu should be classified, flu was perceived homogeneously as belonging to the category of diseases “without severity” and “which can be cured”. The disease was not considered severe, except for “frail people”. The concept of “frail people” evoked a label of people with decreased immune defenses, and belonged to the representation of flu shared by all participants. This category included people of frail constitution (often sick), patients weakened by a disease (cancer, AIDS, asthma), elderly people (aged > 70 years), and young children (aged < 3 years old for parents, and < 10 years old for non-parents). These groups were considered to be the ones where contagion was easiest. However, even if the participant had any of these characteristics, none of them considered themselves as “frail" people.

About two-thirds of respondents were parents, and about 50% lived with their children, among which almost 50% were < 3 years old. These respondents did not differ from those who did not live with children. Several mentioned behaviors to try and avoid contagion that were related to their views of the mode of transmission: in particular, avoiding close contact. Again, many indicated that this might not be realistic in everyday life; they stressed, however, that while they would try to avoid close contact with their children if they were sick, they would not do so if their child was too unwell:

“*With a colleague, I’d avoid staying besides, shake hands, maybe say ‘don’t come to work’ […] If it’s one of my children, I’d take no precautions, because I think, ‘never mind if I get it’, it’s my children, so it’s not like with another person…[If I were sick] then I would not touch them too much or stay away a little bit or, say, even wear a mask, or when I cook, cook for them first and me I’d eat afterwards…*” [respondent 36, woman, 40 years old].

When specifically questioned about vaccination as a means of prevention, few of the respondents indicated that they had ever received the vaccine or were ready to consider it, unless they fell into the category of “frail people”. The study did not specifically address the issue of vaccination and this was not the focus of the interview. However, it is noteworthy that several respondents spontaneously mentioned vaccination as a preventative measure; however , of the total, only five respondents had actually been vaccinated. Of these, two fulfilled the age criterion (> 65 years), which allows for free vaccination in France). The vaccination was often described as "not really useful" (“*[…] it’s very uncertain, I got vaccinated and it didn’t prevent me from catching the flu.*.” [respondent 10, man, 44 years old]), especially for people who were not "frail": “*I was proposed it at my workplace but refused because at my age: it’s not necessary, so I decided not to have it. […] The vaccine is useful for old people because they’re frail people…*” [respondent 16, man, 34 years old]). In some instances it was even considered to be possibly dangerous: “*I think that if you have too much of it… my body might no longer be able to… it may lose the immunity for that and I say to myself that maybe if I have the flu this year, maybe I have more chance to heal than if I catch it when I’m 40 and I’ve had a lot of vaccines… see what I mean?*” [respondent 40, woman, 28 years old].

### Request for healthcare

Patients visited their physician if, and only if, the ILI impaired or persisted after being treated by self-medication with paracetamol and/or homeopathic medicines for 24–48 hours. Self-medication, using over-the-counter drugs, was mentioned in general rather than as an exception to deal with the first symptoms of the continuum described above. However, there was clear concern if a severe fever (> 39°C) developed or severe fatigue that prevented getting out of bed: these factors led them to contact their physician without hesitation. As indicated above, the doctor was consulted to confirm the need for an interruption of activities. In cases where children were affected, the wait was less before consulting a physician, with fever being the key symptom for an immediate appointment. Participants requested that the physician confirmed the patients diagnosis of their child's condition or of their own condition, even if they felt that the physician could do nothing more than what they had done themselves. “*One tries to get better as much as one can and if one doesn’t get any better after 3 days, it’s time to go to the doctor and then it can really be the flu if he says so. It’s the doctor who can say so, it’s not us*” [respondent 2, woman, 47 year old].

Once "flu" has been declared, participants considered that few or even no medications could cure the disease: fatalism was a common reaction. Some people answered categorically that only rest and paracetamol, used against the symptoms and not against the virus, were useful. Many people left it to a physician they trusted. They indicated that they hoped he/she would prescribe an efficient treatment. Antibiotics were only rarely mentioned and, even when mentioned, with little optimism. Rest and paracetamol were identified by the participants as the main possible “remedies”. None of the respondents mentioned anti-viral drugs such as being a potentially active etiological treatment, although these drugs (and in particular oseltamivir) received a lot of attention in the scientific literature and media at the time of the H1N1 pandemic.

Most participants globally expressed little interest in getting more information about "flu". As one participant stated: “*Getting the flu is to be at the wrong place at the wrong moment… (…) it’s not severe, it’s not lethal so I don’t think I need any more information”* [respondent 1, woman, 40 years old]. Furthermore, participants reported that information about flu was sometimes presented as a kind of annually recurring old tune in the media.

## Discussion

This study provides information regarding the representation of flu and ILI and reports the experiences and behaviors of people in relation to influenza within the community. Five major themes were identified. A clear continuum between ILI and flu was described. Flu was considered a very contagious disease, but most subjects stated they had not had flu more than once or twice in their lives. Although the disease was considered benign, most responders identified it as a particular risk for frail people (a category defined for them), but to which they considered they did not belong. The main issue was that flu interrupted daily activities, and this was pathognomonic of influenza for most subjects. In addition, a request for healthcare mainly consisted of confirming a self-diagnosis.

One of the main outcomes of this study, which needs to be underlined, is the homogeneity of the representation of flu: the main dimensions of this representation were similar whatever the age, gender, or socio-professional group of the participants. Flu is constructed as a social object that is recurrently presented in the community, mainly through the media. The "flu" issue re-emerges every year and revolves around the same themes. Whatever its source, the overall message is similar, which results in representations that are very similar to those within the public domain and focus on the same aspects. As this participant summarized: “*It’s all over in the media, the flu, it’s like a chestnut. It’s like the start of the new school year or something like that, at some point in time you hear about it in the media: there it is, “the epidemy is coming”, you Google it and you see how many hits on the flu… one’s immersed in a lot of information on the topic. It comes from all over the place*” [respondent 33, man, 48 years old]. This does not mean that communication about flu should be similar for all population groups: as the study by Gray et al. has shown [9], the ‘one size fits all’ strategy risks reducing trust in agencies and the likelihood that advice will not be followed. Indeed, although the public's representations may be the same (or close), information should be tailored to the needs and expectations of diverse groups [9]. This is important to avoid repetitive information being ignored rather than changing attitudes and behaviors.

Another salient feature of this study was the importance of interruption in daily activities. Sociological studies have underlined the role of interruption in daily activities as a sign of disease or illness [25,26]. Diseases cannot be reduced to their organic aspects: they also involve behaviors, such as interrupting one’s activities, which may change the meaning of a diffuse cluster of somatic symptoms. Interference with normal activities contributes to conferring a disease label to the symptoms, i.e. the symptoms are identified as a disease when daily-life activities are disrupted. Interestingly, the other behavior that was described as contributing to this disease labeling was health care utilization and, more specifically, visiting a physician to get a diagnosis and, thus, to give meaning to the symptoms. The results of the present study point to the role of the physician in being responsible for diagnosing flu, which has been already identified by the individual through a cluster of physical symptoms and interruption of daily activities. Indeed, once flu has occurred, treatment is seen, at best, to reduce the natural course of the symptoms rather than as a cure against a viral infection.

Flu stands out as an apparently paradoxical health condition: although participants described it as very contagious, they also indicated that they had experienced it at most once or twice in their lives, and none perceived this paradox. This may well be related to the continuum from having a “cold” to having “flu”, including “ILI” in-between. Thus, ambivalent physical symptoms may initially develop, but do not specifically indicate the presence of flu itself: the disease may then develop further or the symptoms can disappear. The representation that people hold of flu may then place it in-between being an endogenous entity and an exogenous model [15], where healing is alternatively viewed as a regulating activity and the suppression of a hostile condition, according to the location of symptoms on the above-mentioned continuum.

As rather frequent, the ILI was also seen as indicating a benign condition that does not deserve specific medical attention, except in frail people. This concept raised another paradox: although the notion of frail people was known by all participants, none of the participants considered themselves within this category. This agrees with the results of studies that investigated vaccination in the elderly [5]: the results showed how the perception of a threat guided an individual's behavior. Similarly, at the time of onset of the H1N1 pandemic, an Australian cohort study showed that the vast majority of respondents (> 80%) considered that they had little or no risk of becoming contaminated. Preventive behaviors, such as washing hands, were increased, but only in respondents who considered that they were at risk; in addition, 40% had concerns about the vaccine [27]. Another longitudinal cohort study conducted in Switzerland, before and after the occurrence of the H1N1 pandemic, showed that the perceived threat acted as a predictor of the perceived efficacy of both the vaccine and the preventive measures [28].

Our study has limitations: it was conducted in France and, therefore, the results cannot be transferred to other countries without further investigations. However, in France, due to the sampling methods we used, it may be considered that the dimensions of the representations described in this study are valid for the global population. However, the possible impact of socio-cultural differences could not be investigated due to the French regulation. It has also to be noted that, although subjects were interviewed regarding their representations, no study of their actual behaviors was undertaken.

## Conclusion

Taken together, the results of this study indicate the existence of a gap between the people’s representations and scientific knowledge, i.e.,, between a continuum from having a “cold” to having “influenza (flu)”, including “influenza-like illness” in-between, versus a distinction between “true” influenza and influenza-like illnesses based on the existence of a confirmatory virological diagnosis. This gap in perception makes it more difficult to provide preventative recommendations, as these recommendations may not be implemented when the symptoms are considered moderate. Specifically, the representation of flu as responsible for interrupting daily activities while also seen as a non-severe disease, as well as the perception that it is mainly a threat to “frail people”, but with no individual considering themselves “frail”, is not being addressed in the current campaigns on flu prevention.

In this context, recommendations, such as using a respiratory mask to avoid transmission, may be overlooked (decreased or stopped) when the symptoms are alleviated while the individual is still contagious. The gate thus appears as narrow between a dramatisation of ILI that does not seem to be a proper solution, and an inclusion of the risks related to the characteristics of “true” influenza. Awareness of this narrow gate may allow us to increase the potential benefits of flu-prevention recommendations, both in terms of public health and in daily general medical practice.

## Abbreviation

ILI: Influenza-like illness.

## Competing interests

CC, LS, and FC received honoraria from Laboratoires Boiron for participating in an independent expert board. MFB was an employee of Laboratoires Boiron at the time of the study.

## Authors’ contributions

Conception and design of the study, including semi-structured interview guide: CC, LS, PK, MFB, FC. Data collection: PK. Data analysis: CC, PK. Drafting of the manuscript: CC, LS, FC. All authors reviewed and approved the final manuscript. All authors read and approved the final manuscript.

## Pre-publication history

The pre-publication history for this paper can be accessed here:

http://www.biomedcentral.com/1471-2296/14/15/prepub

## References

[B1] Lin Y Knowledge, Attitudes and Practices (KAP) related to the Pandemic (H1N1) 2009 among Chinese general population: a telephone survey BMC Infect Dis 2011 11 128 10.1186/1471-2334-11-128 21575222 PMC3112099

[B2] Helman C "Feed a cold, starve a fever“- folk models of infection in an English suburban community, and their relation to medical treatment Cult Med Psychiatr 1978 2 107 137 10.1007/BF00054580 81735

[B3] Lee GM Friedman JF Ross-Degnan D Hibberd PL Goldmann DA Misconceptions about colds and predictors of health service utilization Pediatrics 2003 111 231 236 10.1542/peds.111.2.231 12563044

[B4] Gautier A, Jauffret-Roustide M, Jestin C Enquête Nicolle 2006. Connaissances, attitudes et comportements face aux risques infectieux 2008 Saint-Denis: INPES, Collection Etudes santé

[B5] Evans MR Prout H Prior L Tapper-Jones LM Butler CC A qualitative study of lay beliefs about influenza immunisation in older people Br J Gen Pract 2007 57 352 358 17504584 PMC2047008

[B6] Seale H Heywood AE McLaws ML Why do I need it? I am not at risk! Public perceptions towards the pandemic (H1N1) 2009 vaccine BMC Infect Dis 2010 10 99 10.1186/1471-2334-10-99 20403201 PMC2864274

[B7] Morrison LG Yardley L What infection control measures will people carry out to reduce transmission of pandemic influenza? A focus group study BMC Public Health 2009 9 258 10.1186/1471-2458-9-258 19627568 PMC2720966

[B8] Teasdale E Yardley L Understanding responses to government health recommendations: public perceptions of government advice for managing the H1N1 (swine flu) influenza pandemic Patient Educ Couns 2011 85 413 418 10.1016/j.pec.2010.12.026 21295434

[B9] Gray L MacDonald C Mackie B Paton D Johnston D Baker MG Community responses to communication campaigns for influenza A (H1N1): a focus group study BMC Pub Health 2012 12 205 10.1186/1471-2458-12-205 22429559 PMC3324376

[B10] d'Alessandro E Hubert D Launay O Bassinet L Lortholary O Jaffre Y Sermet-Gaudelus I Determinants of refusal of A/H1N1 pandemic vaccination in a high risk population: a qualitative approach PLoS One 2012 7 e34054 10.1371 22506011 10.1371/journal.pone.0034054 PMC3323624

[B11] Carrat F Vergu E Ferguson NM Time lines of infection and disease in human influenza: a review of volunteer challenge studies Am J Epidemiol 2008 167 775 10.1093/aje/kwm375 18230677

[B12] Jodelet D Moscovici S Représentation sociale: phénomènes, concept et théorie Psychologie Sociale 1984 Paris: Presses Universitaires de France 357 378

[B13] Moscovici S Farr RM, Moscovici S The phenomenon of social representations Social representations 1984 Oxford, Paris: Cambridge University Press 3 69

[B14] Horne R Patients' beliefs about treatment: the hidden determinant of treatment outcome? J Psychosom Res 1999 47 491 495 10.1016/S0022-3999(99)00058-6 10661596

[B15] Laplantine F Jodelet D Anthropologie des systèmes de représentations de la maladie Les représentations sociales 2007 7 Paris: PUF 297 318

[B16] Le Breton D La chair à vif. Usages médicaux et mondains du corps humain 1993 Paris: Métailié

[B17] Kuzel AJ Crabtree BF, Miller WL Sampling in qualitative inquiry Doing Qualitative Research 1992 Newbury Park, CA: Sage 33 46

[B18] Mays N Pope Ceds: Qualitative research in health care 1996 London: BMJ Publishing Group

[B19] Schwartz N, Sudman S Context Effects in Social and Psychological Research 1992 New York: Springer-Verlag

[B20] Lincoln Y Guba E Naturalistic inquiry 1985 New York: Sage

[B21] Kenten C Bowling A Lambert N Howe A Rowe G A study of patient expectations in a Norfolk general practice Health Expect 2010 13 273 284 20550590 10.1111/j.1369-7625.2010.00603.x PMC5060535

[B22] Spencer J Ritchie L Bryman A, Burgess RG Qualitative data analysis for applied policy research Analysing qualitative data 1994 London: Routledge

[B23] Strauss A Corbin J Basics of qualitative research: Grounded theory, procedures and techniques 1990 London: Sage

[B24] Pope C Ziebland S Mays N Qualitative research in health care. Analysing qualitative data BMJ 2000 320 114 116 10.1136/bmj.320.7227.114 10625273 PMC1117368

[B25] Herzlich C Santé et maladie - Analyse d'une représentation sociale 2005 Paris: Editions de l'Ecole des Hautes Etudes en Sciences Sociales (3rd edition) [Health and Illness; a Social Psychological Analysis (Translated by Douglas Graham). Paris: Editions de l'Ecole des Hautes Etudes en Sciences Sociales]

[B26] Herzlich C Pierret J Malades d'hier, malades d'aujourd'hui. De la mort collective au devoir de guérison 1991 2 Paris: Payot

[B27] Seale Why do I need it? I am not at risk! Public perceptions towards the pandemic (H1N1) 2009 vaccine BMC Infect Dis 2010 10 99 10.1186/1471-2334-10-99 20403201 PMC2864274

[B28] Gilles I Bangerter A Clémence A Trust in medical organizations predicts pandemic (H1N1) 2009 vaccination behavior and perceived efficacy of protection measures in the Swiss public Eur J Epidemiol 2011 26 203 210 10.1007/s10654-011-9577-2 21476079

